# Depression and Perceived Social Support among Unemployed Youths in China: Investigating the Roles of Emotion-Regulation Difficulties and Self-Efficacy

**DOI:** 10.3390/ijerph19084676

**Published:** 2022-04-13

**Authors:** Zhiya Hua, Dandan Ma

**Affiliations:** 1 School of Government, Shanghai University of Political Science and Law, Shanghai 201701, China; 2 School of Sociology and Political Science, Shanghai University, Shanghai 200444, China; tangdi@shu.edu.cn

**Keywords:** depression, perceived social support, emotion-regulation difficulties, self-efficacy, unemployed youths, unemployment, China

## Abstract

In recent years, the issue of youth unemployment has begun to emerge in China. Unemployed young people are at high risk of depression and other mental health problems. The present study investigates influential factors related to depression and examines the possible mediating effects of difficulties in emotion regulation and self-efficacy between perceived social support and depressive symptoms among unemployed youths in China. Through community recruitment, 511 unemployed young people from Shanghai participated in this cross-sectional survey. The results demonstrate that the prevalence of probable depression in the sample was 49.3% (95% CI: 45.0–53.7%). Moreover, we found that both the perceived social support and self-efficacy were significant negative predictors of depression, whereas difficulties in emotion regulation were positive predictors of depression. In addition, the analysis results indicate that difficulties in emotion regulation and self-efficacy partially mediate the relationship between perceived social support and depression. Overall, this cross-sectional study reveals that depression and mental health problems among China’s unemployed youths are concerning while identifying emotion-regulation difficulties as a risk factor for these and social support and self-efficacy as protective factors, all of which warrant our attention in preventing and intervening with cases of youth depression.

## 1. Introduction

The number of unemployed young people in China has increased significantly in recent years. The International Labour Organization (ILO) defines unemployed youth as young people who meet all of the following criteria: aged between 15 and 24; not in full-time education or training; have the desire and ability to work but cannot find work [1] (p. 22). Because the legal minimum working age in China is 16, China’s unemployed youth refer to those unemployed young women and men aged between 16 and 24. Some of them entered the labor market as soon as they completed nine years of compulsory education (including primary school and junior high school), while others completed secondary vocational or higher education. In the past decades, due to the rapid economic growth of the country, it has not been difficult for China’s young people to find a job. In recent years, however, with the slowdown of economic growth, the shock of the COVID-19 pandemic, and the mismatch of workforce supply and demand caused by the continuous expansion of higher education, more and more young people in China have found that jobs are hard to come by. For example, the Ministry of Education of China reported that about 23% of college graduates cannot obtain a job at graduation [2]. According to China’s National Statistics Bureau, the surveyed unemployment rate for people aged between 16 and 24 rose from 11.9% in 2019 to 14.2% in 2020 and 14.3% in 2021 [3]. In the same years, the surveyed unemployment rates for the 25–59 age group were 4.6%, 5.0%, and 4.5%, respectively [3]. Although the youth unemployment rate in China is slightly below the world average [1] (p. 22), given the country’s huge youth population (nearly 148 million in 2020 [4]), the total number of unemployed young people in China is quite large.

At the individual level, unemployment results from involuntarily losing a job or failing to find a job when entering the workforce [5]. As a stressful life event, becoming unemployed usually entails a sequence of adverse impacts including not only income interruption [6], financial strain [7], erosion of time structure [8], and decline in social status [9], but also worsening of physical and mental health [10,11,12,13]. Numerous studies have pointed out that unemployed people often suffered greater stress and psychological distress than the general population [14,15,16]. Many researchers have found that unemployment, followed by the “chain of adversity” [17], is significantly correlated with a higher risk of depression [18,19,20,21,22]. The World Health Organization (WHO) also warns that “unemployment contributes to and may catalyze the development of depression” [23]. In a meta-analysis, Paul and Moser reported that, compared to the employed, the incidence of depression among unemployed people increases from 16% to 34% [24]. For young people, employment is a sign of successful transition to adulthood in many cultures [25], meaning those who cannot find jobs often suffer additional stresses, frustrations, and social stigma. Young people at transitional ages usually lack sufficient coping resources and strategies [26]. Thus, unemployed youths are seen as a vulnerable age segment in the jobless population [5], and some studies reveal that the young unemployed, compared to their older counterparts, are at higher risk of psychological health disorders, such as depression and anxiety [27,28,29].

Depression is an important health problem that can reduce individuals’ interest and pleasure in life and may trigger the risk of self-injury or suicide [30]. In addition, depression can impair the ability to meet daily demands, the capacity to function well in society, and the competence to handle life challenges [31,32]. For example, studies have reported that depressive symptoms of the unemployed decrease their job-search motivation, intensity, and quality of reemployment [33,34]. These findings indicate that depression not only threatens the physical and psychological well-being of the unemployed but also depreciates and further restricts their job-search performance and reemployment success. Given that the unemployment rate of young people has been significantly higher than that of older adults for many years [1] (p. 13), it is imperative to further reveal the underlying process and mechanisms of depression in the course of unemployment and to seek effective means of protection and intervention to lessen unemployed youths’ depression.

## 2. Literature Review and Research Aims

### 2.1. Depression and Perceived Social Support

As a common mental disorder, depression does not refer to short-term mood fluctuations but a persistent state of negative mood, such as sadness, emptiness, or irritability, which is usually accompanied by somatic and cognitive changes [35]. According to the WHO, the worldwide prevalence of depression is 3.8%, and about 280 million people globally have been affected by depression in recent years [23]. In China, a nationwide survey found the lifetime and 12-month prevalence of depression were 6.9% and 3.6%, respectively [36]. Moreover, studies have revealed that the incidence of depression tends to be young, with many young people in China experiencing an episode [37].

As depression can lead a person toward myriad means of harm, identifying contributing factors and underlying mechanisms has been the focus of considerable research. It is, to date, generally believed that depression is caused by a complicated interaction of biological, psychological and social variables [38]. Substantial research has revealed that depression is significantly correlated with both individuals’ personal characteristics (including demographic and psychological attributes) and external environmental variables (e.g., random events, social ties, and cultural influences) [39]. Social support, as a kind of important resource from others, is on the list of the protective factors of depression, i.e., support from social networks can play the role of buffer between adversities and individuals’ mental health and alleviate the likelihood of depression [40]. Social support refers to the material, emotional, and informational assistance obtained from one’s social networks, which usually reflects the closeness and quality of a person’s connectedness with others [41]. In some academic literature, social support is further classified as received social support and perceived social support [42]. The former emphasizes practical or visible assistance, while the latter highlights the perception of the available resources and subjective experience of being respected, understood, and supported in social relations [43]. Some studies point out that perceived social support as a type of psychological reality can exert more influence than received social support on individuals’ mental health [44]. Considerable research, including both cross-sectional and longitudinal studies, has found perceived social support to be significantly negatively related to depression [45,46,47]. Perceived social support is regarded as helpful for absorbing the impact of adversities and strengthening individuals’ courage and confidence to deal with life’s challenges, and hence, can reduce the risk of depression [48]. For example, one study based on the analysis of old people in Ireland found that perceived social support was a significant negative predictor of later-life depressive symptoms among those once exposed to childhood adversity [49]. On the contrary, lack of perceived social support was found to predict depression [50,51,52]. For instance, two researchers in a study on female twins found the risk of depression was positively associated with tension, disagreements, and criticism in interpersonal relationships [53]. Although a large number of studies conducted in various social groups have tested the link between perceived social support and depression, few studies have examined this relationship among China’s unemployed youths. In addition, the potential mechanisms accounting for the correlation between perceived social support and depression have not been fully explored [54]. Hence, the present study attempted to further examine the underlying process linking perceived social support and depression through a survey of unemployed Chinese youths.

### 2.2. Perceived Social Support, Emotion-Regulation Difficulties, Self-Efficacy, and Depression

In the process of exploring the risk factors of depression, increasing attention has been paid to difficulties with emotion regulation in recent years. Emotion-regulation difficulties were defined as deficits in modulating emotions, especially those for managing negative emotional experiences and expression, thus hindering the formation of adaptative emotions and hence affecting individuals’ mental health [55,56,57]. According to John and Gross, difficulties in emotion regulation are usually embodied in two key interactive processes: emotional insight and behaviors triggered by an emotional response [58]. Specifically, emotion-regulation difficulties include denial of emotion, emotional consciousness defects, and lack of clarity regarding emotion in the first process and impairments associated with the second process, such as a lack of effective emotion-management strategies or ability to control impulses or engage in goal-directed activities when distressed [59,60]. Previous studies have examined the association between emotion-regulation difficulties and some maladaptive behavioral issues (e.g., anxiety, post-traumatic stress disorder, and sleep disturbance) [61,62], and recent research revealed that emotion dysregulation caused by emotion-regulation difficulties, was also significantly positively related to depression [57,63,64,65]. For example, through a survey of a sample of 64 Norwegian adolescents, Visted and colleagues found a lack of positive emotion-regulating strategies and that adopting maladaptive methods, such as rumination and suppression may result in the persistence of a depressive mood and increase the risk of onset or relapse of depression [66]. In comparison, fewer emotion-regulation difficulties were found to be associated with reduced depressive symptoms. For example, an internet-based longitudinal study conducted during the COVID-19 pandemic reported that a decrease in emotion-regulation difficulties can significantly predict a reduction in anxiety and depression [67]. Emotion regulation was proposed to be a context-dependent process that can be affected by environmental variables [68]. Some research found that perceived social support could enhance people’s emotion-modulating abilities and reduce emotion-regulation difficulties [69,70,71]. English and colleagues suggested that strong ties with and more support from others help the emotional adaptation process by encouraging the utilization of such positive emotion-regulation strategies as reappraisal while decreasing the adoption of negative emotion-regulation strategies, such as expression suppression [69]. Moreover, a survey of Venezuelan migrants in Peru confirmed that the perception of support from family members can predict the utilization of reappraisal strategies and decrease emotion dysregulation [72]. Taken together, higher degrees of emotion-regulation difficulties are correlated with higher levels of risk of depressive symptoms but lower levels of perception of support from others. In addition, some studies proposed that emotion-regulation difficulties served as mediators between perceived social support and mental health [70,71]. Based on the above-mentioned correlations, we infer that difficulties in emotion regulation mediate the relationship between perceived social support and depression. However, this mediating model has not been tested by empirical research.

Besides risk factors, protective factors of depression are also a focus of scholars’ attention. A large number of studies have emphasized the protective effect of self-efficacy on depression [73,74]. Self-efficacy refers to one’s belief and confidence in his or her abilities to plan and perform certain actions to achieve desirable goals [75]. According to Bandura, self-efficacy helps individuals positively react to challenges and manage and control their life situations [76]; hence, people with more self-efficacy can cope with adversities calmly and avoid negative impacts on their mental health. This theoretical suggestion has been confirmed by recent research. Some studies have found self-efficacy to be negatively associated with depression [77,78,79,80]. For instance, an empirical study investigated a group of American cancer survivors and found that less self-efficacy was associated with more severe depressive symptoms [74], whereas another study reported that participation in training programs aimed at improving self-efficacy significantly decreased the depression scores of pregnant women in Iran [81]. In addition, some studies suggested that self-efficacy can also be affected by interpersonal processes, such as social interaction and social support [82,83,84,85]. For example, Siciliano argued that self-efficacy can be strengthened by relevant knowledge and beliefs accessed from the individual’s social network [86]. Indeed, there is evidence to support the positive correlation between social support and self-efficacy, i.e., people with a higher level of perceived social support often feel more confident in their abilities [87,88]. Hence, self-efficacy is positively associated with perceived social support while negatively related to depression. Moreover, some studies have empirically confirmed the mediating role of self-efficacy between some environmental variables (e.g., stressful life events, intimate partner violence) and depression [73,89]. Considering all these relationships, self-efficacy may be expected to mediate the link between perceived social support and depression, but few empirical studies have rigorously tested this mediating relationship.

### 2.3. Research Aims and Hypotheses

In summary, although the number of unemployed young people is rising sharply in China and the unemployed youths are at high risk of depression, this group and their mental health problems have not received enough attention. At the same time, substantial studies have linked perceived social support to depression, but the underlying mechanisms between them have not been fully revealed. To fill in these gaps, this study attempted to examine depression and its influential factors among China’s unemployed youths and explore the possible mediating roles of emotion-regulation difficulties and self-efficacy between perceived social support and depression. Specifically, four hypotheses were proposed, as follows: (1) Depression and perceived social support are negatively correlated. (2) Depression is positively associated with emotion-regulation difficulties. (3) Depression is negatively associated with self-efficacy. (4) Emotion-regulation difficulties and self-efficacy act as mediators between perceived social support and depression.

## 3. Materials and Methods

### 3.1. Sampling Process

To test the above-mentioned hypotheses, we conducted a cross-sectional investigation from December 2020 to April 2021 among unemployed youths living in Shanghai, China. The research protocol was examined and approved by the corresponding author’s university. We utilized multi-stage convenience sampling to collect data. First, six districts were selected from the 16 districts in Shanghai. During the second stage, 20 neighborhoods were extracted from each selected district. During the third stage, with the help of neighborhood committees, local social workers, and employment assistants, the research team contacted unemployed young people living in the 120 selected neighborhoods and invited them to participate in the study. Following Hussmanns’ definition of unemployment [90], the research team screened the unemployed young people according to the following enrolment criteria: aged between 16 and 24; no job since leaving school or last job at least one month ago; having been actively seeking and available for jobs within the past four weeks. At this stage, 164 young people refused our invitations and 578 accepted. During the fourth stage, our trained research assistants visited those who accepted the invitations at their homes or places agreed to by them, such as a fast-food restaurant or a meeting room of a local social-work institution. Detailed explanations about the study’s purpose, principles of authenticity, and the researchers’ obligation to protect the participants’ privacy were presented to those young people before the investigation. Only those who gave informed consent were further invited to fill out a questionnaire including some demographic questions and a set of rating scales. The research assistants provided on-site guidance if there were any questions. Eventually, 511 people completed the questionnaires, which constituted our final sample.

### 3.2. Variables and Measures

#### 3.2.1. Demographics

We collected participants’ personal information (age, sex, level of education, etc.) and unemployment experiences (duration, registration status, etc.) through a brief self-report questionnaire.

#### 3.2.2. Perceived Social Support

The perception of support from one’s social networks was assessed by the Multi-Dimensional Scale of Perceived Social Support (MSPSS) developed by Zimet and colleagues [91]. This rating scale consists of 12 self-report items that evaluate the degree of social support from family, friends, and significant others. Responses are given on a seven-point Likert scale for each item (“1” = very strongly disagree, “7” = very strongly agree). The total score is the sum of the scores for each item, ranging from 12 to 84. The higher the total score, the more social support is perceived by people. The Chinese version of the MSPSS has shown good psychometric properties among Chinese people [92,93]. Cronbach’s alpha coefficient of the MSPSS in this study was 0.899, which indicated it had high reliability.

#### 3.2.3. Depression

In this study, we used Beck Depression Inventory-II (BDI-II), designed by Beck and colleagues [94], to evaluate the participants’ depression. The scale includes 21 self-report items that assess the degree of depressive symptoms. Responses are designed on a four-point Likert scale for each item (“0” = not at all, “3” = severely). The total score is obtained by summing the scores of 21 items. The higher the score, the more severe the depression. According to the BDI-II manual, a cut-off point of 13 distinguishes those with (total score ≥ 14) and without (total score < 14) depression. Moreover, a total BDI-II score of 14–19, 20–28, or 29–63 indicates mild, moderate, or severe depression, respectively [94]. BDI-II has been used among the Chinese population and has good psychometric properties [95,96]. Cronbach’s alpha coefficient of BDI-II was 0.889 in the present study, which indicated good internal consistency.

#### 3.2.4. Emotion-Regulation Difficulties

Researchers usually use the Difficulties in Emotion Regulation Scale (DERS), developed by Gratz and Roemer [59], to evaluate emotion dysregulation caused by difficulties with emotion regulation, but this rating scale contains 36 self-report items and so is not suitable for some situations. Bjureberg and colleagues developed a brief version of it, the DERS-16 [97]. Studies have demonstrated that the DERS-16, compared to the DERS, indicates high internal consistency and shows good convergent and discriminant validities [98,99]. To shorten the questionnaire fill-out time, we adopted the DERS-16 in this study. Items in the DERS-16 are completed on a five-point Likert scale (“1” = almost never, “5” = almost always) to assess emotion-regulation difficulties, and the total score is obtained by summing the scores of all items. Higher scores indicate high degrees of difficulties in emotion regulation. The DERS-16 has been translated into Chinese and satisfactory psychometric properties have been indicated for its use in Chinese samples [100]. Cronbach’s alpha coefficient of the DERS-16 was 0.837 in the present study.

#### 3.2.5. Self-Efficacy

We adopted the Generalized Self-Efficacy Scale (GSES), developed by Schwarzer [101], to evaluate participants’ self-efficacy. This scale consists of 10 items to assess optimistic self-beliefs when encountering difficulties. Responses are given on a four-point Likert scale for each item (“1” = completely incorrect, “4” = completely correct). The total score is the sum of scores of all 10 items, and a higher total score indicates a higher sense of self-efficacy. The GSES has been widely utilized by Chinese researchers, and good psychometric properties have been indicated for its use among the Chinese population [102]. The GSES indicated good internal reliability in the current study (Cronbach’s alpha = 0.891).

### 3.3. Statistical Analysis

Data were analyzed using SPSS 26.0 (SPSS Inc., IBM, Chicago, IL, USA). In phase one, we utilized chi-squared tests to analyze whether there were differences in the prevalence of probable depression among unemployed young people with different demographic characteristics. In phase two, Pearson’s bivariate correlation analyses were conducted to test the relationships between perceived social support, emotion-regulation difficulties, self-efficacy, and depression. In phase three, mediation analysis was performed to test the indirect relationships of perceived social support to depression through emotion-regulation difficulties and self-efficacy. As the hypothesized model has two mediators, to effectively examine the multiple mediation effects, the bootstrapping techniques outlined by Preacher and Hayes [103] were adopted and the PROCESS macro for SPSS (version 3.5), designed by Hayes, was utilized. Compared to conventional mediation analysis methods (e.g., causal step regression, Sobel test), the bootstrapping approach need not assume the normal distribution of indirect effects and can test multiple mediators simultaneously [104,105]. Following the advice of Preacher and Hayes [103], all indirect effects were evaluated through bias-corrected 95% confidence intervals based on 5000 bootstrap samples. If the confidence interval did not contain zero, the indirect effect was considered statistically significant.

## 4. Results

### 4.1. Demographic Statistics

All participants in the present study were unemployed young people aged between 16 and 24 (M = 21.51, SD = 2.22). Of the 511 participants, there were more males (61.4%, *n* = 314) than females (38.6%, *n* = 197). Most of the participants were unmarried (87.3%), and local residents of Shanghai (75.3%). More than one-third of the participants (37.8%) completed higher education. In addition, the majority of participants had been unemployed for 12 to 36 months (37.6%), followed by 6 to 12 months (22.5%), and 36 months or more (16.0%), but only 27.6% of them had officially registered their unemployment (Table 1).

### 4.2. Depression Prevalence

In the current study, the prevalence of probable depression was 49.3% (252/511) (95% CI: 45.0–53.7%). More specifically, unemployed young people with probable mild, moderate, or severe depression accounted for 24.1% (*n* = 123), 19.8% (*n* = 101), and 5.5% (*n* = 28) of the overall sample, respectively. Judging from the results of chi-squared tests, the difference in the prevalence of probable depression between each group of the sample was not statistically significant (Table 1).

### 4.3. Correlations between Variables

Table 2 reports the means, standard deviations, and correlation coefficients between all variables of interest. The results demonstrate that perceived social support and depression were significantly negatively correlated (r = −0.305, *p* < 0.001). Moreover, depression was significantly positively related to difficulties in emotion regulation, whereas it was negatively associated with self-efficacy. These correlations provided the basis for the following mediation analysis.

### 4.4. Mediation Analysis

**Figure 1:**
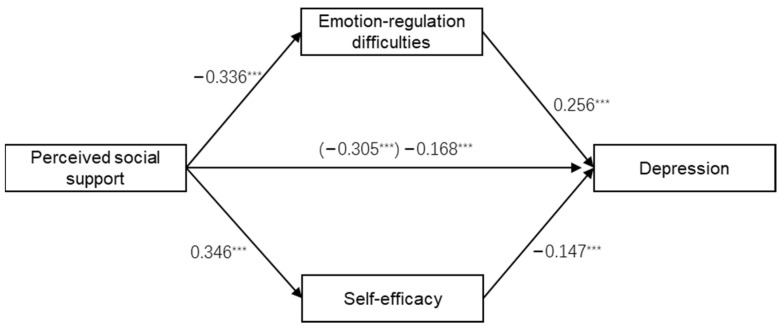
The mediating model for perceived social support, emotion-regulation difficulties, self-efficacy, and depression (N = 511, *** *p* < 0.001).

A multiple mediation model was used to test whether emotion-regulation difficulties and self-efficacy act as mediators between perceived social support and depression. Utilizing the method of bootstrapping estimation with 5000 samples, we first examined the standardized regression coefficient from perceived social support to depression, and the result (β = −0.305, *p* < 0.001) indicated that the total effect was significant. Second, we found the effects of perceived social support on emotion-regulation difficulties (β = −0.336, *p* < 0.001) and self-efficacy (β = 0.346, *p* < 0.001) were significant. Moreover, the paths from emotion-regulation difficulties and self-efficacy to depression were also significant (β = 0.256 and −0.147, respectively). Lastly, when the hypothesized mediators (emotion-regulation difficulties and self-efficacy) were added to the model, we found the direct effect of perceived social support on depression was still significant, but its absolute value decreased from 0.305 to 0.168 (see Figure 1). All these demonstrated that emotion-regulation difficulties and self-efficacy partially mediate the relationship between perceived social support and depression.

In addition, the bootstrapping estimation in the PROCESS macro directly tested the significance of the mediation effects that emotion-regulation difficulties and self-efficacy exert on the link between perceived social support and depression. Using model four in PROCESS macro with 5000 samples, if the 95% confidence interval of the indirect effect outcome did not include zero, the mediation effect was considered to be significant at the level of 0.05. As presented in Table 3, the indirect effects of perceived social support on depression through emotion-regulation difficulties and self-efficacy were significant.

## 5. Discussion

Based on a cross-sectional survey, this study examined probable depression and its influencing factors among unemployed youths in China and tested the possible mediating roles of emotion-regulation difficulties and self-efficacy between perceived social support and depression. The findings demonstrated that the prevalence of probable depression among unemployed young people in China was high. In our sample, the prevalence of probable depression was 49.3%, which was higher than the prevalence among Chinese adolescents (36.6%) [106] and university students (37.0%) [107]. It is important to note that the present study utilized self-report questionnaires instead of rigorous diagnostic interviews to determine depression, which may risk overestimating the prevalence [108]. Although empirical research on depression among China’s unemployed youths is scarce, a survey on unemployed migrant workers in eastern China reported that 51% of the participants suffered from depression [109], which is comparable to the findings of the present study. Therefore, the issue of depression among unemployed youths in China should be considered to be concerning.

Moreover, through Pearson’s correlation analysis, we found significant correlations existed among perceived social support, emotion-regulation difficulties, self-efficacy, and depression among unemployed young people. First, perceived social support and depressive symptoms were significantly negatively correlated. Individuals with more perception of support from their social networks were at a lower risk of depression. This finding added new empirical support to the buffering model originally outlined by Cohen and Wills [41]. Second, a greater perception of social support was significantly related to reduced emotion-regulation difficulties and increased self-efficacy. Third, depression was positively associated with emotion-regulation difficulties and negatively associated with self-efficacy, which indicated emotion-regulation difficulties were the risk factor and self-efficacy was the protective factor of depression. These findings support our corresponding hypotheses.

In addition, mediation analysis demonstrated that, as hypothesized, the mediating effect of emotion-regulation difficulties in the relationship between perceived social support and depression was significant. This finding means unemployed youths with a greater perception of social support usually have a lower level of emotion-regulation difficulties, which may decrease the risk of depression. This result is in line with the findings of previous studies [110]. For example, through an investigation of 902 young adults in the United States, Janelle Welkie and colleagues found that emotion-regulation difficulties mediated the relationship between attention-deficit/hyperactivity disorder and depression [65]. A study of 340 adults with childhood maltreatment experiences in Germany demonstrated that emotion-regulation difficulties partially mediated the relationship between childhood maltreatment and depressive symptoms [111]. Besides these, studies have also confirmed the mediating role of emotion-regulation difficulties among various social groups [112,113]. Through investigating unemployed youths in China, the present study introduced a new empirical sample to test the mediating effects of emotion-regulation difficulties between perceived social support and depression; these could perhaps be explained by the emotion inhibition model [114]. Empirical research has demonstrated that emotion-regulation difficulties can result in chronic emotion inhibition, which, in turn, puts people at a higher risk of psychological disorders, such as depression [112].

Additionally, our analyses revealed that self-efficacy also acted as a significant mediator between perceived social support and depression. This finding implies that increased perceived social support is correlated with enhanced self-efficacy, and in turn, with a decreased risk of depression. This result is consistent with previous findings [115]. For instance, through a survey of 578 men who have sex with men across China, Peng and colleagues reported that self-efficacy partially mediated the link between intimate partner violence and depression [89]. Similarly, in a cross-sectional survey of 305 Chinese caregivers of stroke inpatients, Cong et al. found that self-efficacy exerted a mediating effect on the association between insomnia-related symptoms and depression [116]. The present study provides new empirical support for the mediation effect of self-efficacy, which may be explained by the self-control model [117]. According to this theory, self-efficacy can increase individuals’ abilities to control their psychological and behavioral processes to better adapt to the external environment, which helps reduce the risk of psychological problems [118,119].

The findings of the present study have significant theoretical and practical value. On the one hand, to our knowledge, this study is the first attempt to investigate influential factors related to depression and examine the mediating roles of emotion-regulation difficulties and self-efficacy between perceived social support and depression among unemployed youths in China. The data presented in this study provide empirical support for conceptual work linking social support, emotion-regulation difficulties, self-efficacy, and depression associated with youth unemployment. At the same time, the findings of this study extend our theoretical and empirical understanding of the complicated mechanisms and processes of depression in the course of unemployment. On the other hand, the findings of this study provide a basis for improving intervention programs aiming to reduce depression and improve psychological well-being among unemployed youths. Youth unemployment is a worldwide social issue, and nearly 70 million young people are competing for jobs in the labor market [1] (p. 22). Similarly, youth unemployment is also a crisis in the making in China, and unemployed Chinese young people have not received due attention during the rapid economic growth in the past decades. The findings of the present study indicate that a lower level of perceived social support may exert a negative influence on the psychological health of unemployed youths and increase the risk of depression. On the contrary, greater perceived social support is helpful to absorb the shocks of unemployment and keep a positive mood. Hence, co-building a supportive environment by families, friends, communities, and other social forces, as well as delivering care and assistance to unemployed young people, can help to decrease their risk of psychological disorders, such as depression. Moreover, emotion-regulation difficulties were found to be the risk factor while self-efficacy was the protective factor of depression. Therefore, specific training programs aimed at improving emotion-regulation abilities and self-efficacy can be integrated into intervention projects for unemployed youths, which may help them to effectively cope with a negative mood and stay happy and optimistic during unemployment.

Despite the aforementioned implications, the limitations of this study are also noteworthy. First, this study is based on a cross-sectional survey, which prevents it from making causal statements. Additionally, the cross-sectional research design even risks “the danger of reverse causation” [5]. For example, becoming unemployed can negatively impact young people’s mental health, but it is also possible that unemployment is caused by poor psychological health. To avoid this risk and control the possible selection effect, longitudinal and experimental studies controlling young people’s mental health conditions before they enter into unemployment can be designed in the future. Second, all participants were recruited from Shanghai, the biggest city in China, and local residents made up the majority of the sample (75.3%), which restricts the generalizability of the findings to the larger unemployed population. In the future, more segments of unemployed youths, such as those living in rural areas and unemployed migrant youths, can be investigated. Thirdly, data collection depended exclusively on self-reported measures that were not sufficiently objective. Additionally, this study primarily concerned the impact of unemployment on psychological health while it failed to consider physical health factors. Therefore, further research can promote cross-disciplinary efforts, adopting objective measurements, such as health symptom checklists, diagnostic interviews, and even biochemical indices, to comprehensively and accurately evaluate the health effect of youth unemployment.

## 6. Conclusions

In conclusion, the present study investigated depression and its contributing factors among unemployed youths in China and tested the mediating roles of emotion-regulation difficulties and self-efficacy between perceived social support and depression. The results demonstrated that the prevalence of probable depression among unemployed Chinese youths was high. Moreover, we found that depression and perceived social support were significantly negatively correlated. Meanwhile, emotion-regulation difficulties and self-efficacy partially mediated the relationship between perceived social support and depression. Our findings indicate that depression among unemployed youths is concerning and that perceived social support, emotion-regulation difficulties, and self-efficacy warrant substantial attention in the field of depression prevention and intervention.

## Figures and Tables

**Table 1:** Description of sample demographics and the results of chi-squared tests (N = 511).

Variable	%	N	BDI-II ≥ 14 (*n* = 252)	Depression Prevalence (row%)	*p* (Chi-Squared Test)
Gender					
Male	61.4	314	150	47.8	0.378
Female	38.6	197	102	51.8	
Age					
16–19	21.1	108	49	45.4	0.356
20–24	78.9	403	203	50.4	
Education					
Primary school and below	1.2	6	3	50.0	0.972
Junior high school	20.9	107	51	47.7	
Senior high school (including secondary vocational school)	40.1	205	99	48.3	
College	36.2	185	95	51.4	
Graduate school	1.6	8	4	50.0	
Marital Status					
Unmarried	87.3	446	221	49.6	0.552
Married	12.5	64	30	46.9	
Divorced or others	0.2	1	1	100.0	
Place of household registration					
Shanghai	75.3	385	191	49.6	0.815
Non-Shanghai	24.7	126	61	48.4	
Duration of unemployment					
1 month < ~ ≤ 3 months	8.0	41	24	58.5	0.337
3 months < ~ ≤ 6 months	15.9	81	43	53.1	
6 months < ~ ≤ 12 months	22.5	115	61	53.0	
12 months < ~ ≤ 36 months	37.6	192	85	44.3	
>36 months	16.0	82	39	47.6	
Unemployment registration					
Registered	27.6	141	76	53.9	0.201
Not registered	72.4	370	176	47.6	

Note: The level of significance was set at *p* < 0.05.

**Table 2:** Means, standard deviations, minimum values, maximum values, and correlation coefficients between variables of interest.

Variables	1	2	3	4	Mean	SD	Min	Max	Range
1. PSS	1				46.69	14.85	16	82	12–84
2. ERD	−0.336	1			50.85	7.99	26	74	16–80
3. Self-efficacy	0.346	−0.168	1		26.96	5.76	12	40	10–40
4. Depression	−0.305	0.337	−0.248	1	14.19	8.26	0	48	0–63

Note: SD = standard deviations, Min = minimum values, Max = maximum values, PSS = perceived social support, ERD = emotion-regulation difficulties. All the correlations are significant at the level of 0.001.

**Table 3:** Bootstrapping indirect, direct, and total effects and 95% confidence intervals for the mediation model.

No.	Pathways	Effect Value	95% CI	
			Lower	Upper
1	PSS–RED–depression	−0.0478	−0.0691	−0.0303
2	PSS–self-efficacy–depression	−0.0284	−0.0459	−0.0124
3	PSS–depression (Direct effect)	−0.0936	−0.1429	−0.0443
4	Total effect	−0.1698	−0.2160	−0.1237

Note: CI = confidence intervals, PSS = perceived social support, ERD = emotion-regulation difficulties.

## Data Availability

The data presented in this study are available on request from the corresponding author. The data are not publicly available to preserve the participants’ privacy.
